# Knowledge, attitudes, and practices of parents in rural China on the use of antibiotics in children: a cross-sectional study

**DOI:** 10.1186/1471-2334-14-112

**Published:** 2014-02-27

**Authors:** Miao Yu, Genming Zhao, Cecilia Stålsby Lundborg, Yipin Zhu, Qi Zhao, Biao Xu

**Affiliations:** 1 Key Laboratory of Public Health Safety, Ministry of Education; Department of Epidemiology, School of Public Health, Fudan University, Shanghai, China; 2 Division of Global Health (IHCAR), Department of Public Health Sciences, Karolinska Institutet, Stockholm, Sweden

**Keywords:** Antimicrobial agents, KAP, Self-medication, Medicating children

## Abstract

**Background:**

The purpose of the study was to investigate parents’ perceptions of antibiotic use for their children, interactions between parents and physicians regarding treatment with antibiotics, and factors associated with parents self-medicating children with antibiotics.

**Methods:**

A cross-sectional study was conducted in vaccination clinics in two rural Chinese counties. Primary caregivers (the child’s parents in 97% of cases) visiting these clinics for the vaccination of their young children were given a 55-item structured questionnaire to collect information on the parents’ knowledge and attitudes regarding when, why, and how to use antibiotics and on their practices of purchasing antibiotics and medicating children.

**Results:**

Of the 854 participating primary caregivers, 79% thought antibiotics could cure viral infections, and half believed that antibiotics could shorten the duration of upper respiratory tract infection. Parents reported a median of two hospital visits for their children during the previous 6 months, equal to the median number of antibiotic prescriptions received from physicians. Sixty-two percent of the parents had self-medicated their children with antibiotics. Living in rural villages (Adj OR = 1.643, 95% CI: 1.108–2.436), raising more than one child (Adj OR = 2.174, 95% CI: 1.485–3.183), increasing age of child (Adj OR = 1.146, 95% CI: 1.037–1.266), purchasing antibiotics without a prescription (Adj OR = 6.264, 95% CI: 4.144–9.469), storing antibiotics at home (Adj OR = 2.792, 95% CI: 1.961–3.975) and good adherence to physicians’ advice (Adj OR = 0.639, 95% CI: 0.451–0.906) were independently associated with self-medicating behavior.

**Conclusions:**

Low levels of knowledge on the use of antibiotics and a high prevalence of self-medicating children with antibiotics were observed among parents in rural China. Interventions for the rational use of antibiotics in children should focus on strengthening mass health education, improving effective communication between physicians and patients, and enforcing supervision of the sale of antibiotics in retail pharmacies.

## Background

Globally, young children consume considerable amounts of antibiotics. This is likely caused by their susceptibility to infections, particularly upper respiratory tract infections (URTI) [1-4]. However, it has been demonstrated that most URTI are viral in origin. Consequently, the administration of antibiotics yields minimal benefits. Inappropriate use of antibiotics is one of the major causes of the global emergency of antibiotic resistance [5,6]. The problem of the unnecessary use of antibiotics among children is of special concern in low- and middle-income countries because of the higher prevalence of infectious diseases and shortcomings in hygiene, sanitation, and public health in these contexts [7-11].

Several factors have been suggested to explain the inappropriate use of antibiotics in children: over-prescription by physicians [12,13], easy access to antibiotics for self-medication [14-16], and parents’ limited knowledge about antibiotics [17]. Parents’ perceptions and practices of how to use medicines have important effects on the management of childhood illness [18,19]. If parents enter the parent-physician encounter with the intention of getting antibiotics, this may also bring about the inappropriate use of antibiotics for children through parents’ overt requests or non-overt pressure on physicians [20,21]. In China, especially in rural areas, little is known regarding the association between antibiotic administration to children and parents’ knowledge and attitudes on antibiotic use. This study aims to describe parents’ perceptions of antibiotic use, interactions with physicians for medical treatment, and practices of administering antibiotics to their children. The study also explores the factors contributing to these antibiotic administration practices.

## Methods

### Study area and subjects

A cross-sectional study was carried out in two rural counties of Jiangxi Province in central China. As an indicator of economic activity, the gross domestic profit (GDP) of Jiangxi Province ranks 19^th^ of 31 provinces, municipalities, and autonomous regions in mainland China. A survey was administered in two vaccination clinics of the County’s Center for Disease Control and Prevention. According to the expanded national immunization program, all children living in these areas should go to one of the study clinics for their scheduled vaccinations. Primary caregivers taking children for vaccinations were invited to participate in the survey while their children were under adverse event observation following the inoculation. The study subjects were recruited consecutively in county A, from March 14 through Mar 24, 2012, and in county B, from May 11 through May 25, 2012.

### Data instrument and collection

A 55-item structured questionnaire (Additional file 1) was developed based on a review of the relevant literature [22,23] and the Guideline of Clinical Application of Antimicrobial Agents [24] launched by the Ministry of Health of the People’s Republic of China. Participants were invited to complete the questionnaire independently with detailed guidelines provided for each question. The questionnaire collected information on: (1) demographic characteristics of parents and their children; (2) knowledge and attitudes on antibiotic use, including the basic concepts of antibiotics and indications for their use, methods of administration, and side effects; (3) experiences of interactions with physicians; and (4) practices of purchasing antibiotics without a prescription and of self-medicating children with antibiotics.

A pilot study was conducted among 30 parents at both study sites to optimize the clarity of the self-completion procedure. Based on the pilot study, it was estimated that 35% of parents have a good knowledge of antibiotic use, and a sample size of 743 was calculated, giving a precision of ±10% and type I error of 5% (α = 0.05, Cohen’s *d* = 0.1).

Data collection was completed by four graduate students specializing in epidemiology at Fudan University. These graduate students received training in investigation skills and research ethics in the School of Public Health and from epidemiologists with expertise in antibiotic use and misuse. The four graduate students were responsible for inviting participants and for guiding the completion of the questionnaires following a standardized data collection procedure. The questionnaires were self-administered, and participants completed them individually within 20–30 min.

### Data management and analysis

Questionnaires with responses recorded on more than 90% of the questions were considered well completed and were included in the statistical analysis. All data were double entered by graduate student assistants using Epidata 3.1.0 and were analyzed with SPSS for Windows, Version 16.0 (SPSS, Chicago, IL, USA).

Fourteen questions on the questionnaire were used to assess parents’ knowledge of antibiotics. The proportion answering each question correctly was calculated. To describe the general distribution of awareness, a score of antibiotic knowledge (0–14) was created for each parent based on the number of correct answers to the 14 questions. The median score was used as the cut-off to dichotomize this continuous variable for use as the dependent variable in simple and multiple logistic regression. Parents scoring higher than the median were assessed as having a better knowledge of antibiotic use.

Continuous variables were reported with their median values, and categorical variables were described with frequencies and percentages. Differences in distributions between groups were evaluated using the *χ*^2^ test. Multiple logistic regression models were estimated to examine the associations between parents’ knowledge level of antibiotic use and demographic characteristics, as well as associations between self-medicating children with antibiotics and parents’ sex, age, location, education, medical insurance, age of child undergoing vaccination, number of children, antibiotic knowledge score, attitudes towards self-medication with antibiotics, adherence to physicians’ instructions, experience of purchasing antibiotics without prescription, and experience of storing antibiotics at home.

### Ethical consideration

Informed consent was obtained from all the participating parents in the study. Participants were made aware that they could withdraw at any time. The study was approved by the Institutional Review Board of the School of Public Health at Fudan University.

## Results

### General characteristics of participants

In total, 933 primary caregivers participated in the study, and 854 (92%) completed over 90% of the questionnaire. The 28 primary caregivers (3%) who were grandparents are referred to as “parents” for the purposes of this paper. A summary of the demographic characteristics of the parents and their children is presented in Table 1.

**Table 1:** Demographic characteristics of participants from central towns and villages in Jiangxi Province

**Variables**	**Central towns no. (%)**	**Villages no. (%)**	***P***
Total	481(56)	373(44)	
Gender			.141
Female	362(75)	273(73)	
Male	119(25)	100(17)	
Age of parents, yrs.*			< .001
≤20	10(2)	14(4)	
21-30	272(58)	274(74)	
31-40	161(35)	62(17)	
>40	23(5)	19(5)	
Age of children, yrs.*			.004
<1 (infant)	213(46)	212(58)	
1-6 (preschool)	231(50)	149(41)	
7-12 (elementary)	16(4)	6(2)	
13-15 (junior)	2(0)	0(0)	
Education of parents*			<. 001
Primary school	5(1)	11(3)	
Secondary school	142(30)	229(62)	
High school	144(30)	114(31)	
College or above	185(39)	18(5)	
Number of children*			<. 001
One	296(62)	171(46)	
Two	168(35)	178(48)	
Three	13(3)	22(6)	
Medical insurance*			.183
Social	415(87)	336(91)	
Commercial	7(2)	3(1)	
Social and commercial	14(3)	4(1)	
No insurance	42(9)	28(8)	

*19, 25, 6, 6, and 5 participants had missing values for these variables, respectively.

### Parents’ knowledge and attitudes on antibiotic use

Of the 854 parents responding, 68% believed that they had little knowledge on antibiotic resistance, and 92% wished to be further informed about the appropriate use of antibiotics. The main source for parents to obtain information on antibiotic use was from physicians (71%), followed by pharmacy staff (44%). Compared with parents from rural villages, those living in central towns were more likely to obtain knowledge from books (38 vs. 26%, *χ*^2^ = 12.318, *P* < .001), the internet (35 vs. 22%, *χ*^2^ = 15.772, *P* < .001), and newspapers (16 vs. 9%, *χ*^2^ = 9.429, *P* = .002).

The median score on the 14 questions gauging antibiotic knowledge was 8; and 39% of the parents were assessed as having a better knowledge of antibiotic use. Parents from central towns were significantly more likely than parents from villages to have answered correctly for more than 8 out of the 14 questions (47 vs. 29%, *χ*^2^ = 28.054, *P* < .001) (Table 2). In multiple logistic regression analysis, parents’ education status was the only factor significantly associated with antibiotic knowledge; the higher their education, the better their awareness of antibiotics (Table 3).

**Table 2:** Parents’ responses to questions related to knowledge of antibiotic use

**Questions**	**Correct response**		***P***
	**Central towns no. (%)**	**Villages no. (%)**	
*Basic concepts*			
1. Antibiotics and anti-inflammatory drugs are the same drugs.	180(37)	117(31)	.065
2. Antibiotics can cure infections caused by virus.	102(21)	77(21)	.841
3. Antibiotics should only be obtained with a doctor’ prescription.	309(64)	246(66)	.603
*Indications of antibiotic use*			
4. Antibiotics should be administered in all cases, once a child has fever.	346(72)	247(66)	.072
5. If a child suffers from a cough, running nose, and a sore throat, it will be cured more quickly if he/she receives antibiotic as early as possible.	264(55)	162(43)	.001
6. In most cases, it is not necessary to treat a common cold with antibiotics.	360(75)	241(65)	.001
7. Taking antibiotics in advance can protect children from a common cold.	301(63)	185(50)	< .001
*Administration of antibiotics*			
8. Administration of multiple antibiotics has better effects than a single one.	278(58)	201(54)	.254
9. The more expensive the antibiotic, the more effective it will be.	417(87)	298(80)	.008
10. Antibiotics should be withdrawn as soon as the symptoms disappear.	191(40)	137(37)	.375
*Antibiotic resistance and side effects*			
11. Overuse of antibiotics increases the risk of antibiotic resistance.	326(68)	212(57)	.001
12. Antibiotics do not have side effects.	361(75)	239(64)	.001
13. Scientists can always produce new antibiotics.	89(19)	31(8)	< .001
14. It is dangerous to children if pathogens become resistant to antibiotics.	290(60)	173(46)	< .001

**Table 3:** Factors associated with better knowledge of the uses of antibiotics

**Factors**	**Crude odds ratio (95% CI)**	Adjusted odds ratio* (95% CI)
Female	0.865(0.725-1.031)	0.879 (0.713-1.084)
Age of parents (yrs.)	1.012(0.992-1.032)	1.012 (0.989-1.037)
Living in a village	0.464(0.348-0.618)	0.777 (0.556-1.085)
Education level of parents		
Primary school	0.085(0.019-0.386)	0.089 (0.018-0.426)**
Secondary school	0.212(0.147-0.306)	0.262 (0.170-0.405)**
High school	0.408(0.280-0.594)	0.485 (0.323-0.730)**
College or above	Reference	Reference
With medical insurance	1.161(0.697-1.932)	1.105 (0.645-1.893)
More than one child	0.582(0.440-0.771)	0.873 (0.627-1.217)

*Adjusted odds ratio from multiple logistic regression analysis.

**Statistically significant *P* < 0.05.

A total of 61% of parents believed that antibiotics are overused in China, and 63% agreed that the excessive use increases the risk of antibiotic resistance. Nevertheless, most respondents (86%) thought that scientists can always discover new antibiotics. Seventy-nine percent of parents thought that antibiotics could cure viral infections and half believed that antibiotics could shorten the duration of URTI. A sizeable minority (43%) thought that antibiotics could protect children from the common cold, and these parents were more likely to give their children prophylactic antibiotics than those who knew that antibiotics could not prevent the common cold (30 vs. 11%, *χ*^2^ = 62.852, *P* < .001). Twenty-six percent of parents preferred intravenous infusion of antibiotics over oral administration for their children. Twenty-one percent of parents had not heard of broad-spectrum antibiotics. Another 20% preferred to use broad-spectrum antibiotics, which they thought could kill a great variety of bacteria.

### Interaction between parents and physicians

Parents reported a median of two healthcare visits for their children in the previous 6 months. This is identical to the reported median number of antibiotic prescriptions received from physicians during the same period. About half of these prescriptions were reported to be administered through intravenous infusion. The top three symptoms causing children’s healthcare visits were cough, fever, and running nose.

Of all respondents, 33% recalled that physicians occasionally or never explained the child’s condition or treatment to them. Parents who held a positive evaluation of physicians (reporting that physicians sometimes, often, or always offered an explanation of the child’s condition) were more likely to follow physicians’ advice than those who regarded physicians less positively (reporting that they never or seldom explained the child's condition) (41 vs. 33%, *χ*^2^ = 4.994, *P* = .025).

Forty-five percent of parents considered it reasonable to request antibiotic treatment directly from physicians, and 53% had done this on at least one occasion. Most parents (82%) reported that they would not be dissatisfied if physicians rejected their request for antibiotics, and there was no correlation between the acquisition of antibiotics and parents’ satisfaction with physicians.

### Self-medicating children with antibiotics

In response to the question of whether they had purchased antibiotics without a physician’s prescription on at least one occasion, 40% of parents answered affirmatively. This behavior was more common among parents who did not know that antibiotics should be prescribed by doctors (52 vs. 34%, *χ*^2^ = 26.843, *P* < .001). Three-quarters of parents reported that they had stored antibiotics at home in case of future need, and 62% had medicated their children with antibiotics without the advice of a physician in the previous 12 months.

Among parents who had given their children antibiotics without the advice of a physician in the last 12 months, 55% believed there was no need for medical visits for minor ailments, and 32% admitted using leftover antibiotics that had been previously prescribed by physicians for similar symptoms. A significantly higher proportion of parents from central towns than from villages (19 vs. 12%, *χ*^2^ = 4.566, *P* = .033) considered the convenience of purchasing antibiotics to be a reason for self-medication (Table 4).

**Table 4:** Parents’ reasons for self-medicating children with antibiotics

**Reasons**	**Central towns no. (%)**	**Villages no. (%)**	***P***
1. I thought that my child’s condition was not serious enough	162(56)	131(54)	.728
2. Some antibiotics previously prescribed by physicians for the similar symptoms were left over at home.	92(32)	80(33)	.718
3. It is convenient to purchase antibiotics from retail pharmacies.	56(19)	30(12)	.033
4. I didn’t have enough money to pay for the hospital visit.	32(11)	16(7)	.077
5. I didn’t have enough time to visit a pediatrician.	11(4)	7(3)	.573

After adjusting for potential confounding factors using multiple logistic regression, living in villages (adjusted odds ratio (Adj OR) = 1.643, 95% confidence interval (CI): 1.108–2.436), raising more than one child (Adj OR = 2.174, 95% CI: 1.485–3.183), having ever purchased antibiotics without a prescription (Adj OR = 6.264, 95% CI: 4.144–9.469), having stored antibiotics at home (Adj OR = 2.792, 95% CI: 1.961–3.975), and parents' good adherence to physicians’ advice (Adj OR = 0.639, 95% CI: 0.451–0.906) were significantly associated with self-medication of children with antibiotics. The likelihood of parents self-medicating children with antibiotics increased with the age of the child (Adj OR = 1.146, 95% CI: 1.037–1.266) (Table 5).

**Table 5:** Factors associated with parents’ behavior of self-medicating children with antibiotics

**Factors**	**Crude odds ratio (95% CI)**	Adjusted odds ratio* (95% CI)
Female	1.078(0.916-1.268)	1.048(0.867-1.268)
Age of parents, yrs.	1.015(0.994-1.036)	1.005(0.978-1.033)
Living in villages	1.207(0.911-1.600)	1.643(1.108-2.436)**
Education		
Primary school	0.607(0.218-1.694)	0.191(0.049-0.754)
Secondary school	1.738(1.220-2.476)	1.072(0.636-1.807)
High school	1.260(0.868-1.828)	1.012(0.619-1.654)
College or above	Reference	Reference
Has medical insurance	1.165(0.707-1.920)	0.941(0.515-1.719)
Age of children, yrs.	1.214(1.106-1.332)	1.146(1.037-1.266)**
More than one child	2.595(1.936-3.479)	2.174(1.485-3.183)**
Score of questions on knowledge of antibiotics	0.956(0.909-1.005)	1.002(0.938-1.071)
Believes it is reasonable to self-medicate children with antibiotics.	2.243(1.608-3.127)	1.158(0.759-1.768)
Would be dissatisfied if physicians rejected their request for antibiotics	1.339(0.918-1.952)	1.259(0.784-2.023)
Would follow all the advice from physicians	0.743(0.562-0.983)	0.639(0.451-0.906)**
Once purchased antibiotics without physicians’ prescription	8.469(5.868-12.222)	6.264(4.144-9.469)**
Sometimes, often or always stores antibiotics at home	4.345(3.230-5.845)	2.792(1.961-3.975)**

*Adjusted odds ratio from multiple logistic regression analysis.

**Statistically significant *P* < 0.05.

## Discussion

### Parents’ insufficient and incorrect knowledge on antibiotics

Parents in our study had considerable misunderstandings that may contribute to inappropriate antibiotic use. We found that 79% of parents thought that antibiotics could cure infections caused by viruses, a higher percentage than was found in a pan-European study (54%) [25]. Further, half of the parents in the present study believed that antibiotics could shorten the duration of URTI symptoms, similar to the findings of a study conducted in Cyprus (48%) [26] but higher than previous findings in the United States (19%) [27]. A major concern is that nearly half of the parents in the present study thought that taking antibiotics in advance could protect children from the common cold.

Our findings also suggest a considerable contrast in perceptions of antibiotics between parents living in central towns and those living in rural villages. Specifically, parents from central towns had a better knowledge of appropriate indications and of side effects of antibiotic use. Thus, parents living in rural villages should receive more health education, and village doctors could play an important role in the dissemination of knowledge on the management of childhood infections.

### Communication gap between parents and physicians

Although half of the parents in this study reported that they had requested antibiotics directly from a physician at least once, most parents said they would not feel dissatisfied if their requests were rejected. This discrepancy may indicate that what the parents really seek in their requests is not the antibiotic itself but rather communication with physicians about their children's conditions through sharing the children’s experiences of antibiotic administration.

Evidence from different settings has shown that patients’ satisfaction is less related to obtaining antibiotics and more related to a feeling that physicians have listened to and understood their concerns [28,29]. This can also be demonstrated from our findings that parents who obtained guidance more frequently from physicians had a stronger adherence to physicians’ advice. Conversely, ineffective communication between patients and physicians may lead to the unnecessary prescription of antibiotics. We found that one-third of respondents complained that physicians seldom or even never explained their children’s illness during healthcare visits. Several studies of the patient-physician encounter have reported limited interactions between physicians and patients, mainly owing to the lack of time to talk facing the long line of outpatients waiting or to the lack of a regulated procedure for assisting patients in understanding the disease and treatment [23,30]. Our findings demonstrate the important role of physicians in the appropriate use of antibiotics in rural China, illuminating the urgent need for communication training and professional education among physicians.

### High prevalence of self-medicating children with antibiotics

We found that a high proportion (62%) of parents had self-medicated their children with antibiotics. This finding is much higher than the results of previous studies, which found, for example, that 36% of parents in an urban area of China [15], 12% of parents in suburban areas of Greece [31], and 23% of parents in urban areas of Greece [32] had self-medicated their children with antibiotics.

The reasons for the high prevalence of self-medicating children with antibiotics in our study are multifactorial. Having purchased antibiotics from retail pharmacies without a physician’s prescription at least once is a critical factor contributing to self-medicating children with antibiotics. As Morgan et al. [33] pointed out, poor regulation of antibiotics commonly results from the absence of enforcement of policies. Although the purchase of antibiotics without a prescription is forbidden by State Food and Drug Administration regulations [34], customers nevertheless have easy access to antibiotics in most areas of China. In our study, this phenomenon was more prevalent in central towns than in villages; more parents from central towns regarded the convenience of purchasing antibiotics from retail pharmacies as a reason for self-medication. In practice, parents living in central towns were also more likely to purchase antibiotics from pharmacies without a prescription than were parents living in villages. This may be because the large-scale chain pharmacies are mainly located in populous areas, and parents in central towns can obtain antibiotics from retail pharmacies more easily compared with those in rural areas. In an ongoing European Commission granted project “An integrated syndromic surveillance system for infectious disease in rural China (ISSC),” we found, through the surveillance of medicine sales in retail pharmacies, that some pharmacies used promotional sales incentives to encourage customers to buy two units of some specific brand of drug, including antibiotics, by offering an attractive advertising cup or other small gifts. Over the 2-week promotion, sales of antibiotics, in addition to over-the-counter medications, increased dramatically. This provides further evidence of the purchase of non-prescribed antibiotics in rural China [35].

Storing antibiotics at home also increases the probability of medicating children with antibiotics, a significant portion of home-stored antibiotics were reported to be left over from previous prescriptions. A global survey found that living in a country where antibiotics are dispensed in fixed-count packs rather than as the exact numbers of pills required is a strong predictor for possession of leftover antibiotics [14]. Stopping treatment earlier than prescribed is another source of leftover antibiotics. In our study, two-fifths of parents believed that antibiotics should be withdrawn as soon as the symptoms disappeared, a practice which increases the risk of relapse and the development of resistant pathogens.

### Methodological considerations

This cross-sectional study was carried out among parents who brought their children for vaccination under the expanded immunization program. Of the parents in our study, 29% were new mothers or fathers with babies younger than 1 year old. As these parents have little experience with children’s illness and treatment, the frequency of self-medicating children with antibiotics might be underestimated in this study. Another limitation of the study is that self-reported answers about medicating children with antibiotics will inevitably include a degree of recall bias.

This study was carried out in only two of the more than 2500 counties in China, and limitations to generalizability are inevitable. Nonetheless, our findings are applicable to parents of children undergoing routine immunization. Considering the general development and health indicators in these counties, we expect findings from this study to be relevant to populations in similar settings, especially in poor and less developed regions.

In summary, our findings suggest that insufficient knowledge and incorrect perceptions about antibiotics are prevalent among parents in rural China. Easy access to antibiotics, habits of antibiotic storage at home, and poor adherence to physicians' advice were associated with parental behavior of antibiotic self-medication of children. Furthermore, communication gaps exist between parents and physicians during children’s healthcare visits.

## Conclusions

Improvement in the appropriate use of antibiotics in rural China will require multi-sectoral cooperation and long-term efforts. Health education and interventions for parents could be conducted through physicians, mass media, and community approaches. The contents of health education should include the basic concepts of antibiotics, the appropriate indications and administration, and the potential hazards of medicating children with antibiotics. Physicians play a key role in health education, as they were the main source of information on antibiotics for the respondents in our study. Strategies for effective communication with patients and the prudent prescription of antibiotics should be included in physician education to increase patients’ adherence to advice and consequently to reduce self-medication with antibiotics. Stringent implementation of regulations concerning non-prescribed antibiotics in retail pharmacies is essential to restrict access to antibiotics for self-medication. Completion of the full course of prescribed antibiotics and the discontinuation of use of leftover antibiotics in the home should also be advocated among the general public.

## Competing interests

The authors declare that they have no competing interests.

## Authors’ contributions

MY participated in the design of the cross-sectional study; performed the acquisition, analysis, and interpretation of the data; and drafted the manuscript. BX carried out the design of the study, participated in the manuscript revision, and coordinated the process of language editing. GZ and QZ participated in the design and helped to draft the manuscript. CSL helped to revise the manuscript critically for important intellectual content and gave final approval of the version to be published. YZ participated in the design of the study and helped with the acquisition of the data. All authors read and approved the final manuscript.

## Pre-publication history

The pre-publication history for this paper can be accessed here:

http://www.biomedcentral.com/1471-2334/14/112/prepub

## Supplementary Material

Additional file 1 Questionnaire. Click here for file
