# Supplementary material for: Knowledge, attitudes, and practices of parents in rural China on the use of antibiotics in children: a cross-sectional study
Source: BMC Infect Dis. 2014 Feb 27;14:112. doi: 10.1186/1471-2334-14-112 (PMC3938908; doi:10.1186/1471-2334-14-112)
Supplement: Additional file 1 — Questionnaire. [file 1471-2334-14-112-S1.doc]

**Additional file 1**

Title: Questionnaire for the knowledge, attitudes, and practices of parents in rural China on the use of antibiotics in children

**Questionnaire for the knowledge, attitudes, and practices of parents in rural China on the use of antibiotics in children***

Dear parents,

Good day. We are the graduate students from School of Public Health at Fudan University. We are dedicated to improving the appropriate use of antibiotics among children. Through the survey, your response will help us have a better knowledge of the perceptions and practices of parents in rural China on the use of antibiotics in children. We would appreciate it if you could share with us your opinions and suggestions on this issue. Your response is anonymous and will only be used for survey purposes. Thank you very much for your time and cooperation.

**Demographic characteristics**

1. Your sex: Male; Female

2. Your date of birth: _____(mm/yyyy)

3. Your educational status_______:

(1). Primary School or below

(2). Secondary School

(3). High School/ Secondary technical college

(4). College /University

(5). Postgraduate studies or above

4. Educational status of your spouse:________.

(1). Primary School or below

(2). Secondary School

(3). High School/ Secondary technical college

(4). College /University

(5). Postgraduate studies or above

5. Are you living in:

Central town; Village

6. Your medical insurance (You can choose more than one)________.

(1).Basic medical insurance for township employees

(2).Medical insurance for township residents

(3).New rural cooperative medical insurance

(4).Private insurance

(5).None

(6).Others（please indicate）__________________

7. Medical insurance of your spouse (You can choose more than one)_________.

(1).Basic medical insurance for township employees

(2).Medical insurance for township residents

(3).New rural cooperative medical insurance

(4).Private insurance

(5).None

(6).Others（please indicate）__________________

8.Date of birth of your child for today’s vaccination:_______(mm/yyyy)

9.Sex of your child for today’s vaccination: Male； Female

10.Is the child for today’s vaccination your only child?

Yes (Please go directly to Q12);

No

11. How many children do you raise now: ____

12. There are___ residents in your household. (Resident should have lived in your household more than half year)

13. Would you describe the yearly income of your household in 2011 as: (the yearly income should be the total gross income of all residents in your household, including salary, bonus, business and agricultural income, pension, dividend, interest, rent and any other income)

(1). Below 3,000 RMB

(2). 3,000-4,999 RMB

(3). 5,000-9,999 RMB

(4). 10,000-19,999 RMB

(5). 20,000-49,999 RMB

(6). 50,000 RMB and above

**Section A Please answer the following** questions on knowledge of antibiotics

| Your opinion on the following questions: | Disagree | Agree | Not known |
| --- | --- | --- | --- |
| 14. Antibiotics and anti-inflammatory drugs are the same drug. |  |  |  |
| 15. Antibiotics could cure the infections caused by virus. |  |  |  |
| 16. Antibiotic should be administered in all cases, once a child has fever. |  |  |  |
| 17. Antibiotics do not have side effects. |  |  |  |
| 18. Scientists can always produce new antibiotics. |  |  |  |
| 19. If a child suffers from a cough, running nose, and a sore throat, he/she will be cured more quickly if he/she receives antibiotic as early as possible. |  |  |  |
| 20. Antibiotics should be withdrawn as soon as the symptoms disappear. |  |  |  |
| 21. Overuse of antibiotics increases the risk of antibiotic resistance. |  |  |  |
| 22. Antibiotics should only be obtained with a doctor's prescription. |  |  |  |
| 23. In most cases, it is not necessary to treat a common cold with antibiotics. |  |  |  |
| 24. Administration of multiple antibiotics has better efficacy than that of single one. |  |  |  |
| 25.Taking antibiotics in advance can protect children from a common cold. |  |  |  |
| 26.The more expensive the antibiotic, the more effective it will be. |  |  |  |
| 27. It is dangerous to children if pathogens become resistant to antibiotics. |  |  |  |

28. Which of the following drugs are antibiotics? (You can choose more than one and please tick the appropriate box)

(1). Amoxicillin (6). Dioctahedral smectite（Smecta）

(2). Paracetamol（Tylenol） (7). Norfloxacin

(3). Aspirin (8). Ambroxol（Mucosolvan）

(4). Roxithromycin (9). Compound liquorices tablet

(5). Cephradine

29. Sources of information you have about judicious antibiotic use (you can choose more than one, and please tick the appropriate box):

(1). Physician (2). Pharmacist (3). Friend or family relative

(4). Internet (5). Television (6). Newspaper

(7). Lecture (8). Broadcast (9). Others (please indicate) ________

**Section B Please answer the following questions on your attitudes on** antibiotic use

| Your opinion on the following questions: | Strongly disagree | Disagree | Agree | Strongly agree | Uncertain |
| --- | --- | --- | --- | --- | --- |
| 30. I have little knowledge of bacterial resistance. |  |  |  |  |  |
| 31. I believe antibiotics are used too much in our Country. |  |  |  |  |  |
| 32. Parents should be further informed about judicious use of antibiotics. |  |  |  |  |  |
| 33. I could decide which antibiotics my child should receive according his/her condition. |  |  |  |  |  |
| 34. I should get my child to take antibiotics in prevention, once other children around catch cold. |  |  |  |  |  |
| 35. Pediatricians should confirm the cause of illness according to physical or laboratory examination before prescribing antibiotics for my child. |  |  |  |  |  |
| 36. If my child gets sick, I prefer him/her to receive intravenous infusion of antibiotics instead of oral administration. |  |  |  |  |  |
| 37. I prefer to choose antibiotics which is more expensive. |  |  |  |  |  |
| 38. I should be in compliance with pediatricians’ advice and it’s not appropriate to make further request. |  |  |  |  |  |
| 39. In case I wish my child to receive antibiotics, I will be dissatisfied if the pediatrician refused my request for antibiotics. |  |  |  |  |  |
| 40. I think that my knowledge on appropriate use of antibiotics has been enough. |  |  |  |  |  |
| 41. I prefer to use broad-spectrum antibiotics such as Cefixime, etc., because they could kill a variety of bacteria. |  |  |  |  |  |

**Section C Please answer the following questions on your practice on antibiotic use**

42. Could you recall that in the last half year (from September 1st), how many times did your children visit a pediatrician (vaccination excluded)? _______.

43. In the above-mentioned visits, how many times did pediatricians prescribed antibiotics for your child? _____

44. How many times did your child receive the treatment of intravenous infusion of antibiotics? ­­______

45. Could you recall the respective causes of the last 3 visits in the past half year? (Select from the listed options, and supplement other causes, if any, in the **Others** box.)

|  | Cough | Running nouse | Sore throat | Fever | Rash | Diarrheal /vomit | Headache | Others |
| --- | --- | --- | --- | --- | --- | --- | --- | --- |
| Last time |  |  |  |  |  |  |  |  |
| Penultimate time |  |  |  |  |  |  |  |  |
| Antepenultimate time |  |  |  |  |  |  |  |  |

46. Have you ever purchase antibiotics without physicians’ prescriptions?

Yes; No (please go directly to Q48)

47. Where do you usually purchase antibiotics without physicians’ prescriptions? (You can choose more than one)

(1). Retail pharmacy (2). Online pharmacy

(3). Bazaar (4). Others (please indicate) __________

48. Could you recall that in 2011, how often would you give your child antibiotics without pediatricians’ advice?

(1).Always

(2).Most of the times

(3).Often

(4).Occasionally

(5).Never（please go directly to Q50）

49. For which reasons you would give your child antibiotics without pediatricians’ advice? (You can choose more than one)

(1). I didn’t have enough time to visit a pediatrician.

(2). I thought that my child’s condition was not serious enough.

(3). Some antibiotics previously prescribed by physicians for the similar symptoms were left over at home.

(4). It is convenient to purchase antibiotics from retail pharmacies.

(5). I didn’t have enough money to pay the hospital visit.

(6). Others (please indicate) ___________________

| The number from 0 to 9 represent the frequency of following activities. *Zero* represents “never” and *9* represents “always”. Please tick the appropriate number which can represent your frequency of the activity. | Never | Occasionally | | Often | | | | Most of the times | | Always |
| --- | --- | --- | --- | --- | --- | --- | --- | --- | --- | --- |
| 50. In case you strongly wish your child to receive antibiotics, how often do you request for it directly from the pediatrician? | 0 | 1 | 2 | 3 | 4 | 5 | 6 | 7 | 8 | 9 |
| 51. How often do you follow all the pediatrician’s instructions and advice? | 0 | 1 | 2 | 3 | 4 | 5 | 6 | 7 | 8 | 9 |
| 52. How often do you give your child antibiotics with dosage less than drug instruction in consideration of safety? | 0 | 1 | 2 | 3 | 4 | 5 | 6 | 7 | 8 | 9 |
| 53. How often do you store antibiotics at home in case of future need? | 0 | 1 | 2 | 3 | 4 | 5 | 6 | 7 | 8 | 9 |
| 54. How often do you give your child antibiotics with dosage more than drug instruction in consideration of efficacy? | 0 | 1 | 2 | 3 | 4 | 5 | 6 | 7 | 8 | 9 |
| 55. How often does the pediatrician explain to you about your child’s condition? | 0 | 1 | 2 | 3 | 4 | 5 | 6 | 7 | 8 | 9 |

**Thank you for your cooperation and we wish you and your family all the best!**

*: The questionnaire is developed by researchers in the School of Public Health, Fudan University, Shanghai, China.
